# Cost-effectiveness analysis of the single-implant mandibular overdenture versus conventional complete denture: study protocol for a randomized controlled trial

**DOI:** 10.1186/s13063-016-1646-0

**Published:** 2016-11-04

**Authors:** Túlio Eduardo Nogueira, Shahrokh Esfandiari, Cláudio Rodrigues Leles

**Affiliations:** 1 School of Dentistry, Federal University of Goiás, Avenida Universitária Esquina com 1ª Avenida, s/n. Setor Universitário, Goiânia, Goiás CEP 74605-220 Brazil; 2 Faculty of Dentistry, McGill University, 2001 McGill College Avenue, suite 500, Montreal, QC H3A 1G1 Canada

**Keywords:** Cost-effectiveness, Economic evaluation, Dental economics, Overdentures, Complete dentures, Patient satisfaction

## Abstract

**Background:**

Preliminary clinical studies on the single-implant mandibular overdenture (SIMO) have reported favorable results as an alternative to the conventional complete dentures for rehabilitation of the edentulous mandible. Clinical and patient-reported outcomes were assessed but no evidence is available with respect to the cost-effectiveness of this treatment, which is particularly important to test whether the incremental cost associated with the implant treatment is justified facing the benefits from the intervention. Thus, the aim of this study is to assess the cost-effectiveness of single-implant mandibular overdentures.

**Methods/design:**

This randomized clinical trial will include edentulous individuals who meet eligibility criteria. Participants will be randomized into one of the treatment groups: a conventional complete denture group or a single-implant mandibular overdenture group. Direct costs related to therapies in both groups will be identified, measured and valuated for 1 year after treatment. Oral health-related quality of life and satisfaction with the dentures will be the primary outcome variables. Incremental cost-effectiveness ratios will be estimated and graphically presented on cost-effectiveness planes. A Markov decision tree will be constructed to set out the consequences of the competing alternatives. Sensitivity analysis on the most important assumptions will be performed in order to assess the robustness of the model.

**Discussion:**

This is the first trial-based cost-effectiveness study on single-implant mandibular overdentures. Specific challenges in designing the protocol are considered. The expected results are of high clinical relevance and may contribute to the decision-making process when choosing between different alternatives for the rehabilitation of the edentulous mandible.

**Trial registration:**

ClinicalTrials.gov Identifier: NCT02710357, registered on 11 March 2016.

**Electronic supplementary material:**

The online version of this article (doi:10.1186/s13063-016-1646-0) contains supplementary material, which is available to authorized users.

## Background

Tooth loss is an important public health problem that affects different population groups, having a greater incidence in older people within disadvantaged communities [1, 2]. In Brazil, 53.7 % of the 65–74 year-old group do not have any teeth according to a national survey held in 2010 [3] and the conventional tissue-supported complete denture is still the most common clinical treatment. Although most edentulous patients appear to benefit from complete dentures and report satisfactory oral comfort and masticatory function, it is fairly frequent to find patients who are poorly adapted to their dentures, even with dentures that are perceived to be prosthodontically acceptable [4]. Individuals who experience difficulties with their dentures usually complain of pain, discomfort, poor oral coordination and limited functioning, mainly caused by low retention and stability and an unfavorable condition of the supporting tissues associated with the mandibular denture.

Proper management of unstable and uncomfortable dentures is very challenging, even for experienced prosthodontists. In most cases, the use of dental implants to retain a mandibular denture is recommended to assure a more favorable prognosis for those difficult-to-treat edentulous patients. The mandibular overdenture, retained by two implants in the interforaminal region, has been the standard of care for the edentulous mandible [5, 6]. Available evidence shows that it leads to better outcomes compared to the conventional denture, such as increased patient satisfaction and oral health-related quality of life (OHRQoL) [7]. However, there is no reliable evidence on the ideal number of implants needed to retain a mandibular overdenture [8], and the reduction of the number of implants to a single implant for denture retention has been proposed as a less invasive and less costly alternative compared to other overdenture designs [9].

The single-implant mandibular overdenture (SIMO) was originally recommended for older edentulous patients experiencing discomfort and functional difficulties with conventional mandibular dentures [10]. The implant is placed in the symphyseal region where favorable bone quality and quantity is usually found. This ensures satisfactory primary stability and high implant success rates, even for immediately loaded implants [11]. In addition, this less invasive and less costly implant intervention might allow more people to benefit from this treatment, even with general health concerns.

Previous clinical studies have shown that SIMO significantly improves patient satisfaction and improves OHRQoL impacts when compared to a conventional mandibular denture [12, 13]. Favorable results regarding several other relevant outcomes, such as higher implant survival rates, minimal marginal bone loss and acceptable incidence of adjustments and repairs, were also reported [9–18].

From an economic perspective, the available evidence strongly supports the view that mandibular overdentures retained by two or four implants are cost-effective when financers are willing to pay in excess of the calculated cost-effectiveness acceptance thresholds, considering that willingness to pay may vary considerably between financers pay within and between health care systems and outcome measures [19, 20]. The availability of simpler interventions, like SIMO, could increase the demand for implant overdentures by reducing the potential risks of surgical interventions and the incremental costs associated with the treatment in comparison to overdenture designs with greater number of implants.

The aim of this study is to assess the cost-effectiveness of the SIMO. The study hypothesis is that SIMO is more effective but more costly than the conventional denture, though this incremental cost is relatively low for the offered effectiveness in terms of clinical and patient-reported outcomes.

## Methods/design

This study is a randomized clinical trial. Study groups will include patients who are receiving a complete maxillary denture as opposed to either a conventional mandibular denture (CD – control group) or a SIMO (SIMO – experimental group). Although the two-implant mandibular overdenture is established as the standard of care for the edentulous mandible [5, 6], in most parts of the world the CD is still considered the most common treatment for edentulous patients. Therefore, we chose the CD as the reference strategy to SIMO to test the incremental effect of placing a single implant in the symphyseal region for denture retention.

**Fig. 1:**
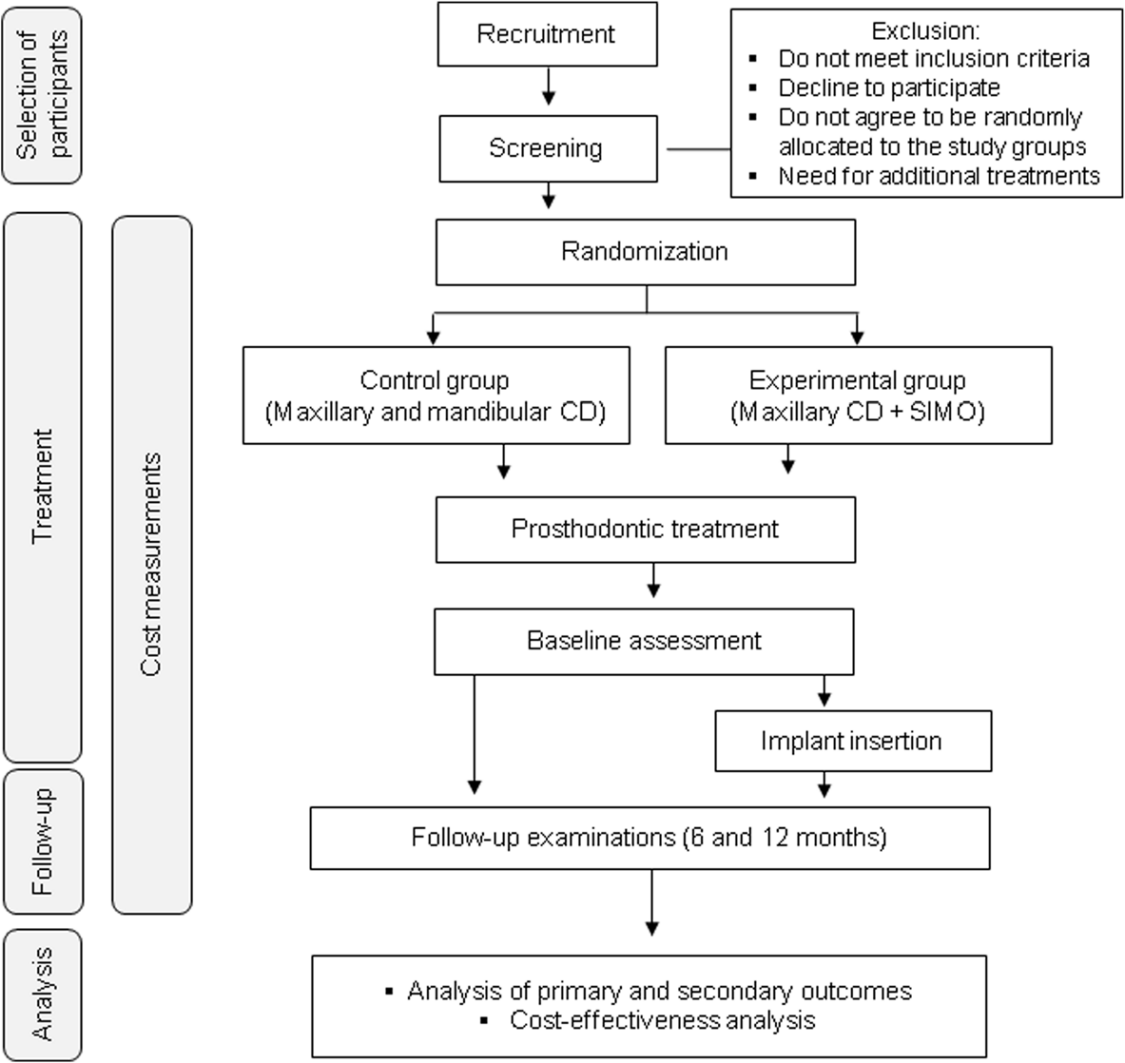
Flowchart of the study

A flowchart of the study is detailed in Fig. 1. The final study protocol was approved by the Research Ethics Committee of the Federal University of Goiás in September 2014 (protocol number: 020/2012). Two documents related to the reporting of clinical trials and health economic evaluations were used to guide the structure of this protocol: the Consolidated Standards of Reporting Trials (CONSORT Statement) [21] and the Consolidated Health Economic Evaluation Reporting Standards (CHEERS) [22] (see Additional file 1). All clinical procedures will be performed at the School of Dentistry of the Federal University of Goiás, Goiânia, Goiás, Brazil. Participants will not be charged for any treatment costs.

### Participants

#### Target population and sample

The target population will comprise fully edentulous subjects. The eligible participants will receive maxillary and mandibular conventional complete dentures. The primary list of participants will be retrieved from the database of registered patients in the local public health system. For the initial screening, no restrictions regarding gender and age will be considered. Treatment needs will be based on normative criteria and/or on subject’s expressed need for replacement of old dentures.

#### Inclusion and exclusion criteria

Referred subjects will be evaluated by means of a thorough clinical assessment and a panoramic radiograph. For inclusion in the study, the screened potential participants must have no contraindications for implant surgery (mainly related to uncontrolled systemic diseases) and present enough bone volume in the mandibular midline area for an implant length of at least 8 mm without the need for bone augmentation procedures. They should be able to understand and answer the questionnaires used in the study and agree to participate by providing a written informed consent.

Noncompliant participants will be excluded from the study. Moreover, individuals who do not agree to be randomly allocated to the treatment study group (CD or SIMO) will be excluded. Subjects who present signs of untreated temporomandibular disorders or uncontrolled systemic or oral conditions that require additional treatments will be referred for proper treatment and be excluded from the study.

#### Stopping criteria

A participant will be removed from the trial in case of withdrawal of the consent or discontinuation of the prescribed treatment program (deviate from protocol); for example, if the participant decides to have other implant treatment modalities during the follow-up period (such as overdentures with different number of implants and/or fixed implant dentures). Any condition preventing the attendance to follow-up visits will be considered as a participant dropout, such as incapacity due to advanced physical debilitation or moving to another city. Interrupted follow-up due to withdraw and/or dropout will be documented and consent will be sought from participants to retain data collected up to the point of withdrawal/dropout.

### Sample size estimation

In this study, sample size was estimated for each of the two treatment groups considering a minimum power of 0.80 (type II error rate) at a two-sided 0.05 significance level (type I error rate). A previous single-group clinical trial that assessed the incremental effect of the SIMO treatment compared to the CD, and considered the outcome satisfaction with the dentures and OHRQoL, served as the data source for this sample size estimation [23]. A minimum of 24 patients will enter this two-treatment parallel-design study, considering that the true difference between treatments is 7.4 units for OHIP-EDENT and 2.6 units for satisfaction scores, assuming unequal variances for both groups. An increase of 10 % to reduce the impact of loss to follow-up on study power resulted in a minimum final sample of 28 participants, 14 in each treatment group.

### Randomization

A computer-based random number generator will be used to assign participants into the treatment groups [24]. In order to prevent imbalance between groups, participants will be stratified according to gender [25]. Block randomization will be used to randomly assign participants to sets of different sizes, using unsorted numbers with a range from 1 to 2 (representing the two treatments) and an allocation ratio of 1:1 for the control and experimental groups. The sequence will be concealed in opaque, consecutively numbered envelopes for each block. After each enrollment, the next randomization envelope will be opened, according to the previously established sequence. An independent collaborator will manage the allocation and inform participants about their assigned groups.

If a participant allocated to the SIMO group refuses to receive the implant treatment (noncompliance), follow-up will occur the same way as in the control group. Moreover, intention-to-treat analysis will be performed, which means that during statistical analysis noncompliant participants will be kept in their original group, even if they did not receive the allocated treatment. This may provide a more conservative estimation of treatment effect, minimizing the probability of type I error occurrence [26].

### Masking/blinding

To avoid selection bias and ensure adequate allocation concealment, their treatment group will only be revealed for each participant after the assessment of the baseline outcomes, which will occur after the delivery and regular use of the new set of conventional dentures. Since full blinding for the two interventions is not possible for those involved with treatment management and collection of data, only those collecting and analyzing clinical data from masticatory performance tests will be unaware of the assigned treatment.

### Interventions

#### Control group (complete denture treatment)

New maxillary and mandibular dentures will be fabricated following a standardized protocol. Clinical steps will include: (1) anamnesis and preliminary impression with alginate (Jeltrate Dustless, Dentsply, Brazil) using stock trays, (2) final impression with a custom tray and zinc oxide eugenol impression material (Lysanda, Lysanda Produtos Odontológicos Ltda., São Paulo, Brazil), (3) occlusal registrations and mounting in a semiadjustable articulator fixed in average settings, without the use of a facebow, (4) a try-in visit to assess the artificial teeth (Trilux, Dental Vip Ltda, Pirassununga, São Paulo, Brazil), (5) delivery of the dentures and (6) visits for adjustment. All laboratory procedures will be carried out by the same dental technician using standard procedures for complete denture processing.

Moreover, the dentures will be technically evaluated by an experienced prosthodontist in order to guarantee maximum quality according to each case. When needed, retreatment or any adjustment/repair in the dentures will be performed accordingly. Participants will receive regular maintenance for their dentures, including adjustments for elimination of sore areas, denture relining and repair of fractures, when needed, until the end of the follow-up period.

#### Experimental group (single-implant mandibular overdenture (SIMO))

##### Prosthodontic phase

All prosthodontic procedures for construction of conventional maxillary and mandibular dentures will be performed similarly to the control group.

##### Presurgical procedures

After insertion and adjustment of the new dentures, implant placement surgery will be planned with the aid of panoramic and lateral cephalometric radiographs, which will serve to determine implant dimensions and position, as well as to assess the conditions of the implant bone site. A cephalometric radiograph will be acquired with the dentures in situ and, to make the proposed implant position evident, a thin strip of lead foil will be fixed outlining the outer surface of the lower denture in the midline position [11]. Imaging exams should confirm the presence of sufficient bone volume for the placement of an implant with a length of at least 8 mm.

In order to assess general health conditions, blood tests will be required, including complete blood count, prothrombin time, activated partial thromboplastin time and fasting blood glucose. In cases of abnormal findings, the participant will be referred to the physician in order to treat the condition before having the implant insertion surgery done.

##### Surgical and prosthodontic procedures

Antibiotic prophylaxis with 2 g of amoxicillin will be administered 1 h preoperatively [27]. In case of allergy to penicillin, 600 mg of clindamycin will be used. Each patient will then receive a Straumann® Standard Plus SLActive® regular neck implant (Straumann 0.33.051S/052S/053S, Institute Straumann AG, Basel, Switzerland) in the mandibular midline. The implant length will be defined according to the bone height availability, which will be assessed by means of the panoramic and cephalometric radiographs, with the aid of a transparent template. After infiltration anesthesia, incision will be performed using a size 15C disposable scalpel blade. Surgical access will be as conservative as possible, involving a minimal crestal incision, except when a more extensive access is needed for regularization of the alveolar bone crest at the discretion of the surgeon. Drilling sequence and implant insertion will be executed according to the manufacturer’s protocol. The upper denture will serve as a reference guide for the installation of the implant and it will be properly disinfected before surgery with 2 % chlorhexidine digluconate by being soaked in this solution for 10 min [28].

Bone quality and jaw shape will be noted according to the classification proposed by Lekholm and Zarb [29]. Furthermore, final insertion torque will be recorded from the drilling unit with the aid of a manual torque wrench. Implant stability quotient (ISQ) will be assessed using a magnetic resonance frequency device (Osstell Mentor, Integration Diagnostics, Göteborg, Sweden). Final insertion torque will be kept around 20 N.cm and the implant platform placed at least 1 mm above the gingival margin. A healing abutment will be connected and the implant will be allowed to heal for 3 weeks. Sutures will be performed with simple interrupted stitches and removed after 7 days. In addition, the mandibular denture will be relined with a soft acrylic temporary relining material (Soft Comfort, Dencril, São Paulo, Brazil).

Postoperatively, participants will be advised to have a soft food diet and to rinse with a 0.12 % chlorhexidine mouthwash for 1 week. Additionally, paracetamol 750 mg will be provided as the analgesic option to be taken to a maximum of four times daily as needed, since it has been shown to be effective in relieving postoperative pain and has a low incidence of adverse effects [30]. Additional postoperative medication will be prescribed for case-specific situations.

After 3 weeks, the healing abutment will be removed, the gingival collar will be irrigated with 0.12 % chlorhexidine and implant stability will be assessed. Subsequently, a 3.4-mm retentive titanium anchor abutment (Straumann 048.439, Institute Straumann AG, Basel, Switzerland) will be connected and tightened to 35 N.cm with a torque wrench. The temporary relining material will be removed and a space relief will be made in the inner part of the denture to accommodate the abutment and the corresponding elliptical matrix (Straumann 048.456, Institute Straumann AG, Basel, Switzerland). The matrix will be incorporated to the denture using self-curing acrylic resin and the patient will be instructed to keep the upper and lower dentures firmly occluded in the habitual position until the final polymerization of the resin. Occlusion will be checked and, if necessary, adjustments will be made to guarantee a balanced occlusal scheme. No interventions will be performed to the maxillary denture.

### Outcomes

The effectiveness of both treatments will be measured by means of two primary patient-centered outcomes: oral health-related quality of life (OHRQoL) impacts and satisfaction with the dentures:

- OHRQoL impacts: the cross-culturally adapted Brazilian version of the Oral Health Impact Profile for edentulous subjects (OHIP-EDENT) will be used [31, 32]. It contains 19 items divided into four different subscale domains: (I) masticatory discomfort and disability (four items), (II) psychological discomfort and disability (five items), (III) social disability (five items) and (IV) oral pain and discomfort (five items). The items are answerable on a 3-point Likert scale and responses will be summed to obtain an overall OHIP-EDENT score and for each of the four domains. Higher scores represent worse OHRQoL
- Satisfaction with the dentures: a 10-cm uninterrupted Visual Analog Scale will be used in order to assess the participants’ ratings of their satisfaction with the upper and lower dentures in relation to the parameters “general satisfaction,” “comfort,” “stability,” “aesthetics,” “ability to speak,” and “ability to chew.” Each participant will indicate their level of satisfaction with each parameter by marking a point along the scale, in which one end signifies “unsatisfied” and the other end signifies “satisfied,” respectively. Then, an assistant will convert each mark into a value of between 0 to 100 by placing a transparent template with numbered intervals from 0 to 100 mm.

Masticatory performance will be assessed as a secondary outcome using a two-colored chewing gum test and a qualitative and quantitative colorimetric method to measure the color-mixing ability [33]. The selected test food will be the Vivident Fruitswing “Karpuz/Asai Üzümü” (Perfetti van Melle, Turkey), a two-colored gum comprising a green and a dark violet layer measuring 43 mm × 12 mm × 3 mm. Participants will be asked to sit upright in a dental chair and to chew the gum on their preferred chewing side for 20 and 50 cycles. The chewed gum will be then retrieved from the oral cavity, placed in a transparent plastic bag and flattened to a 1-mm thick wafer by pressing on a custom-made polyvinyl chloride plate with a milled depression of 1 mm × 50 mm × 50 mm. Each specimen will be labeled with random numbers to allow blind assessment. Both sides of the specimen will be scanned using a flatbed scanner at a resolution of 500 dpi. Visual and electronic analysis of the chewed gum samples will be done according to the classification proposed by Schimmel et al. [33]. Analysis will be made by two investigators blinded to the study groups and treatment stage. ViewGum software (dHAL Software, Kifissia, Greece, www.dhal.com) will be used for electronic colorimetric analysis.

Other clinical outcomes will be recorded in order to provide a comprehensive assessment of the intervention, such as implant survival rate, implant stability quotient, peri-implant bone changes, peri-implant soft tissue aspects and incidence of prosthodontic repair/adjustment events. Control variables, such as age and gender, as well as baseline clinical variables will be recorded for patient description and subgroup data analysis. Subjects will also be classified according to the American College of Prosthodontists’ Index [34].

Table 1 shows all the study outcomes and the time frame for assessment throughout the clinical trial.

**Table 1:** Standard Protocol Items: Recommendations for Interventional Trials (SPIRIT) diagram

	Study period						
	Enrollment	Allocation	Post allocation				
Time point	Pretreatment	*t*_0_	Baseline	Implant placement	*t*_1_	*t*_6_	*t*_12_
Enrollment:							
Eligibility screen	x						
Informed consent	x						
Allocation		x					
Interventions:							
Conventional denture (control group)		x	x			x	x
Single-implant mandibular overdenture (SIMO) (intervention group)		x	x	x	x	x	x
Assessments:							
Oral health-related quality of life (OHRQoL)			x			x	x
Satisfaction with dentures			x			x	x
Masticatory performance			x			x	x
Implant stability^a^				x	x		x
Peri-implant bone level^a^					x	x	x
Peri-implant soft tissue health^a^					x	x	x
Prosthodontic events							
Costs							

^a^Assessed only for the single-implant mandibular overdenture (SIMO) group

### Economic analysis

#### Study perspective

A cost-effectiveness analysis will be performed from the health care provider perspective. Cost estimations of the study interventions will be derived from the clinical trial, which is a reproduction of a typical private setting for oral health care in Brazil, since implant treatment is not covered by the public health system and has limited reimbursement from insurance companies. An additional analysis will be performed using the patient perspective, in which costs will be estimated according to the reference pricing of dental procedures and treatments by the Brazilian Dental Association (Classificação Brasileira Hierarquizada de Procedimentos Odontológicos – CBHPO) and reference gross costs will be derived from a panel of specialists.

#### Time horizon and discount rates

The time frame for primary data collection will be 1 year starting from group assignment of each participant of the clinical trial. For modeling-based data, a maximum time horizon of 10 years will be assumed as a relevant time frame for clinical trials, both to accommodate the needs of decision-makers and to provide a “trajectory” of summary measures over time [35]. In this case, both costs and outcomes will be discounted at an annual rate of 5 %, as recommended by Brazilian guidelines [36].

#### Estimation of costs

Cost estimation will include all expenditures and resources associated with the alternative interventions during the 1-year follow-up period. Relevant costs will be grouped into dental costs (items used for treatment) and nondental costs (e.g., transportation fares). Some assumptions were made for the inclusion and valuing of relevant resource items: (1) no capital costs will be considered, (2) societal costs related to broader costs to society (e.g., productivity losses resulting from treatment, family costs, dietary adaptation, etc.) will not be included.

Cost estimation will involve three stages: identification of relevant resource items used for treatment associated with the provision of the interventions; estimation of the amount of resources used (in natural units); and application of prices (unit costs) to each of the resource items. Table 2 details the parameters that will be used in cost analysis, according to the treatment groups.

**Table 2:** Description of items included in cost analysis, quantification, sources and references for item valuing for the two alternative treatments

Resource items	Cost calculation	Cost estimation method	Source for resource valuing	Estimated for treatment groups	
				CD	SIMO
Dental direct costs					
Overall clinical time					
Denture fabrication	(Monthly wage/monthly working time) × time spent	Time in min	Reference salary	Yes	Yes
Implant surgery time	(Monthly wage/monthly working time) × time spent	Time in min	Reference salary	No	Yes
Overdenture time	(Monthly wage/monthly working time) × time spent	Time in min	Reference salary	No	Yes
Follow-up	(Monthly wage/monthly working time) × time spent	Time in min	Reference salary	Yes	Yes
Implant	Units × price per unit	Microcosting	Price catalog	No	Yes
Prosthodontic components	Units × price per unit	Microcosting	Price catalog	No	Yes
Medication	Units × price per unit	Microcosting	Reference price	No	Yes
Imaging exams	Units × price per unit	Gross-costing	Mean market prices	Yes	Yes
Surgical consumables	Overall consumable resources/cases	Microcosting	Mean market prices	No	Yes
Prosthodontic laboratory	Units × price per unit	Gross-costing	Reference prices	Yes	Yes
Blood tests	Overall cost of exams per patient	Gross-costing	Reference prices	No	Yes
Consumables					
Infection control and PPE	Overall consumable resources/cases	Microcosting	Mean market prices	Yes	Yes
Dental materials	Overall consumable resources/cases	Microcosting	Mean market prices	Yes	Yes
Instruments/equipments					
Conventional denture	Estimation of lifespan for each item	Estimated annual costs	Panel of specialists	Yes	Yes
Implant/overdenture	Estimation of lifespan for each item	Estimated annual costs	Panel of specialists	No	Yes
Nondental direct costs					
Transportation fares	Number of visits per patient	Self-reported	Cost from public transportation	Yes	Yes

*CD* conventional mandibular denture, *PPE* personal protective equipment, *SIMO* single-implant mandibular overdenture

Overall costs for the two alternative treatments will include the summative value of all direct costs listed in Table 2. The currency used for cost estimations will be Brazilian reals – BRL R$. Price date will be recorded and values will be adjusted to the year of the registered costs by applying specific price indexes. If currency conversion is required for the final report, purchasing power parity (PPP) will be used to equalize the purchasing power of different currencies by eliminating the differences in price levels between countries. Alternatively, a widely used currency, such as the US dollar, will be adopted for cost estimations.

### Data analysis

#### Descriptive and inferential statistics

Data will be checked and cleaned blind to the treatment group allocation and analysis will be carried out according to the intention-to-treat principle. Statistical analysis will comprise between-group comparisons of the effectiveness outcomes (OHIP and patient satisfaction) and will be performed using independent *t* tests or nonparametric tests in case of asymmetric distribution of data (IBM-SPSS 23.0 software; IBM Corp, 2015). An alpha level of 0.05 will be set for significance.

#### Cost-effectiveness analysis

A cost-effectiveness analysis will be carried out by calculating the incremental cost-effectiveness ratio (ICER), which represents a single, common effect that measures the difference in magnitude between the two alternatives using data retrieved from a clinical trial, expressed in terms of the incremental cost per unit of effect, as follows:

$$ ICER=\left(Cos{t}_a-Cos{t}_b\right)/\left( Effectivenes{s}_a- Effectivenes{s}_b\right), $$

where the measures of effectiveness will be considered to be the satisfaction with the dentures and the OHRQoL impacts.

Decision analysis will be carried out to model the outcomes of decision between the two alternatives. This analysis considers that the outcomes of decisions are not certain, but that the probabilities of different outcomes are known from the trial-based data. The Markov model with Monte Carlo simulation will be used since the decision analysis problem involves risk that is continuous over time, the timing of events is important, and important events may happen more than once.

Markov models assume that a patient is always in one of a finite number of discrete health states, called Markov states [37]. All events are represented as transitions from one state to another. Important events for this study will be represented in a tree-type diagram that identifies the decision alternatives (decision node), the list of the possible outcomes of each alternative (chance nodes) and the sequence of events (terminal node). The full decision tree will contain all the relevant nodes and the paths between them, as well as the probabilities assigned to chance events and the values attached to each potential final state. Data derived from the clinical trial will be used to construct a Markov decision-tree model of CD versus SIMO, considering the most relevant clinical events, their related probabilities of transitions from one state to another) and estimated costs from each course of action.

One-way sensitivity analysis will be performed to assess the impact that changes in certain parameters (e.g., implant/component costs, time horizon, etc.) will have on the model’s conclusions, as well as to determine which parameters are the key drivers of a model’s results. A tornado diagram will be constructed to show graphically the results of various assumptions on the final outcome in the sensitivity analysis. TreeAge Pro 2014 software will be used for the cost-effectiveness analysis.

## Discussion

This is the first trial-based cost-effectiveness study on SIMOs. Specific challenges in designing the protocol are considered. The expected results are of high clinical relevance and may contribute to the clinical decision-making process, offering a novel approach for the treatment of mandibular edentulism.

The scarcity of economic analysis in prosthodontics has major practical implications, since clinical decision-making might occur without there being reliable evidence relating to the costs and consequences of prosthodontic care. Therefore, the best possible decision cannot always be achieved in the scenario of limited financial resources [38]. Another critical challenge to conduct cost-effectiveness analysis in prosthodontics is to incorporate validated clinical outcomes that can be used to compare and contrast costs of the intervention [38]. This study protocol suggests two commonly used patient-reported measures for edentulous subjects: a validated instrument for assessment of OHRQoL impacts and a multi-item measure of patient satisfaction with the mandibular denture. The definition of resource items, criteria for cost calculation and sources for item valuing are also proposed.

The incorporation of implants as part of oral rehabilitation alternatives also gives rise to important issues regarding health economics. Since many implant interventions involve relevant costs for both the patient and prosthodontist, the search for a more efficient use of available resources to achieve better benefits with lower costs is mandatory in many clinical situations. A systematic literature review [39] on economic aspects of implant interventions, focusing on cost-effectiveness of dental implants compared with other conventional treatment options, showed that replacement of multiple teeth with dental implants was associated with higher initial costs along with greater improvements in patient-reported outcomes. However, there is an evident lack of studies dealing with measurement of costs and consequences in specific clinical scenarios involving edentulous subjects and implants. This is both surprising and disappointing because this type of study is essential due to concern about the increase in health care expenditure associated with limited resources to meet the demands of both patients and society [40].

Finally, from an economic perspective, it is important to bear in mind that, in most societies, dental care is not fully incorporated as an integral part of general health services. Hence, there is a predominance of private dental care based on the fee-for-service payment system, in which dentists are paid for every service that they provide based on the usual and customary fees charged in the local area. This probably explains why there is scarce evidence derived from economic studies in dentistry [41, 42] and reinforces the need for economic evaluations of dental interventions, in order to improve decision-making processes and contribute to the development of health care policies.

## Trial status

The trial started recruitment in March 2016.

## Additional file

Additional file 1: CHEERS checklist. (DOC 113 kb)

## Abbreviations

CD: Conventional denture
ICER: Incremental cost-effectiveness ratio
ISQ: Implant stability quotient
OHIP-EDENT: Oral Health Impact Profile for Edentulous
OHRQoL: Oral health-related quality of life
PPP: Purchasing power parity
SIMO: Single-implant mandibular overdenture
