# Supplementary material for: Cost-effectiveness analysis of the single-implant mandibular overdenture versus conventional complete denture: study protocol for a randomized controlled trial
Source: Trials. 2016 Nov 4;17:533. doi: 10.1186/s13063-016-1646-0 (PMC5097429; doi:10.1186/s13063-016-1646-0)
Supplement: Additional file 1: — CHEERS checklist. (DOC 113 kb) [file 13063_2016_1646_MOESM1_ESM.doc]

Consolidated Health Economic Evaluation Reporting Standards – CHEERS Checklist 1

**CHEERS Checklist**

**Items to include when reporting economic evaluations of health interventions**

The **ISPOR CHEERS Task Force Report**, *Consolidated Health Economic Evaluation Reporting* *Standards (CHEERS)—Explanation and Elaboration: A Report of the ISPOR Health Economic Evaluations Publication Guidelines Good Reporting Practices Task Force,* provides examples and further discussion of the 24- item CHEERS Checklist and the CHEERS Statement. It may be accessed via the *Value in Health* or via the ISPOR Health Economic Evaluation Publication Guidelines – CHEERS: Good Reporting Practices webpage: http://www.ispor.org/TaskForces/EconomicPubGuidelines.asp

| **Section/item** | **Item** | **Recommendation** | **Reported** |
| --- | --- | --- | --- |
|  | **No** |  | **on page No/** |
|  |  |  | **line No** |
| **Title and abstract** |  |  |  |
| Title | 1 | Identify the study as an economic evaluation or use more |  |
|  |  | specific terms such as “cost-effectiveness analysis”, and |  |
|  |  | describe the interventions compared. | 01 |
| Abstract | 2 | Provide a structured summary of objectives, perspective, |  |
|  |  | setting, methods (including study design and inputs), results |  |
|  |  | (including base case and uncertainty analyses), and |  |
|  |  | conclusions. | 02 |
| **Introduction** |  |  |  |
| Background and | 3 | Provide an explicit statement of the broader context for the |  |
| objectives |  | study. |  |
|  |  | Present the study question and its relevance for health policy or |  |
|  |  | practice decisions. | 03 |
| **Methods** |  |  |  |
| Target population and | 4 | Describe characteristics of the base case population and |  |
| subgroups |  | subgroups analysed, including why they were chosen. | 05 |
| Setting and location | 5 | State relevant aspects of the system(s) in which the decision(s) |  |
|  |  | need(s) to be made. | 05 |
| Study perspective | 6 | Describe the perspective of the study and relate this to the |  |
|  |  | costs being evaluated. | 14 |
| Comparators | 7 | Describe the interventions or strategies being compared and |  |
|  |  | state why they were chosen. | 4-5 |
| Time horizon | 8 | State the time horizon(s) over which costs and consequences |  |
|  |  | are being evaluated and say why appropriate. | 14 |
| Discount rate | 9 | Report the choice of discount rate(s) used for costs and |  |
|  |  | outcomes and say why appropriate. | 14 |
| Choice of health | 10 | Describe what outcomes were used as the measure(s) of |  |
| outcomes |  | benefit in the evaluation and their relevance for the type of |  |
|  |  | analysis performed. | 11-12 |
| Measurement of | 11a | *Single study-based estimates:* Describe fully the design |  |
| effectiveness |  | features of the single effectiveness study and why the single |  |
|  |  | study was a sufficient source of clinical effectiveness data. | 11-12 |


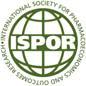


|  | Consolidated Health Economic Evaluation Reporting Standards – CHEERS Checklist 2 | | |
| --- | --- | --- | --- |
|  |  |  | |
|  | 11b | *Synthesis-based estimates:* Describe fully the methods used for | N/A |
|  |  | identification of included studies and synthesis of clinical | |
|  |  | effectiveness data. | |
| Measurement and | 12 | If applicable, describe the population and methods used to | N/A |
| valuation of preference |  | elicit preferences for outcomes. | |
| based outcomes |  |  |  |
| Estimating resources | 13a | *Single study-based economic evaluation:* Describe approaches | 14-16 |
| and costs |  | used to estimate resource use associated with the alternative | |
|  |  | interventions. Describe primary or secondary research methods | |
|  |  | for valuing each resource item in terms of its unit cost. | |
|  |  | Describe any adjustments made to approximate to opportunity costs. | |
|  |  |  | |
|  | 13b | *Model-based economic evaluation:* Describe approaches and | N/A |
|  |  | data sources used to estimate resource use associated with | |
|  |  | model health states. Describe primary or secondary research | |
|  |  | methods for valuing each resource item in terms of its unit | |
|  |  | cost. Describe any adjustments made to approximate to | |
|  |  | opportunity costs. | |
| Currency, price date, | 14 | Report the dates of the estimated resource quantities and unit | N/A |
| and conversion |  | costs. Describe methods for adjusting estimated unit costs to | |
|  |  | the year of reported costs if necessary. Describe methods for | |
|  |  | converting costs into a common currency base and the | |
| Choice of model | 15 | exchange rate. | 14-15 |
|  |  | Describe and give reasons for the specific type of decision- | |
|  |  | analytical model used. Providing a figure to show model | |
|  |  | structure is strongly recommended. | |
| Assumptions | 16 | Describe all structural or other assumptions underpinning the | 17-18 |
|  |  | decision-analytical model. | |
| Analytical methods | 17 | Describe all analytical methods supporting the evaluation. This | 17-18 |
|  |  | could include methods for dealing with skewed, missing, or | |
|  |  | censored data; extrapolation methods; methods for pooling | |
|  |  | data; approaches to validate or make adjustments (such as half | |
|  |  | cycle corrections) to a model; and methods for handling | |
|  |  | population heterogeneity and uncertainty. | |
| **Results** |  |  | 18 |
| Study parameters | 18 | Report the values, ranges, references, and, if used, probability | |
|  |  | distributions for all parameters. Report reasons or sources for | |
|  |  | distributions used to represent uncertainty where appropriate. | |
|  |  | Providing a table to show the input values is strongly | |
|  |  | recommended. | |
| Incremental costs and | 19 | For each intervention, report mean values for the main | N/A |
| outcomes |  | categories of estimated costs and outcomes of interest, as well | |
|  |  | as mean differences between the comparator groups. If | |
|  |  | applicable, report incremental cost-effectiveness ratios. | |
| Characterising | 20a | *Single study-based economic evaluation:* Describe the effects | N/A |
| uncertainty |  | of sampling uncertainty for the estimated incremental cost and | |
|  |  | incremental effectiveness parameters, together with the impact | N/A |


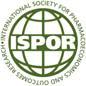


|  | Consolidated Health Economic Evaluation Reporting Standards – CHEERS Checklist 3 | | |
| --- | --- | --- | --- |
|  |  |  | N/A |
|  |  | of methodological assumptions (such as discount rate, study | |
|  |  | perspective). | |
|  | 20b | *Model-based economic evaluation:* Describe the effects on the | N/A |
|  |  | results of uncertainty for all input parameters, and uncertainty | |
| Characterising | 21 | related to the structure of the model and assumptions. | N/A |
|  |  | If applicable, report differences in costs, outcomes, or cost- | |
| heterogeneity |  | effectiveness that can be explained by variations between | |
|  |  | subgroups of patients with different baseline characteristics or | |
|  |  | other observed variability in effects that are not reducible by | |
|  |  | more information. | |
| **Discussion** |  |  | N/A |
| Study findings, | 22 | Summarise key study findings and describe how they support | |
| limitations, |  | the conclusions reached. Discuss limitations and the | |
| generalisability, and |  | generalisability of the findings and how the findings fit with | |
| current knowledge |  | current knowledge. | |
| **Other** |  |  | 21 |
| Source of funding | 23 | Describe how the study was funded and the role of the funder | |
|  |  | in the identification, design, conduct, and reporting of the | |
| Conflicts of interest | 24 | analysis. Describe other non-monetary sources of support. | 21 |
|  |  | Describe any potential for conflict of interest of study | |
|  |  | contributors in accordance with journal policy. In the absence | |
|  |  | of a journal policy, we recommend authors comply with | |
|  |  | International Committee of Medical Journal Editors | |
|  |  | recommendations. | |

For consistency, the CHEERS Statement checklist format is based on the format of the CONSORT statement checklist

The **ISPOR CHEERS Task Force Report** provides examples and further discussion of the 24-item CHEERS Checklist and the CHEERS Statement. It may be accessed via the *Value in Health* link or via the ISPOR Health Economic Evaluation Publication Guidelines – CHEERS: Good Reporting Practices webpage: http://www.ispor.org/TaskForces/EconomicPubGuidelines.asp

The citation for the CHEERS Task Force Report is:


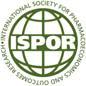
Husereau D, Drummond M, Petrou S, et al. Consolidated health economic evaluation reporting standards (CHEERS)—Explanation and elaboration: A report of the ISPOR health economic evaluations publication guidelines good reporting practices task force. Value Health 2013;16:231-50.
